# Development of Biodegradable Polyesters: Study of Variations in Their Morphological and Thermal Properties through Changes in Composition of Alkyl-Substituted (ε-DL) and Non-Substituted (ε-CL, EB, L-LA) Monomers

**DOI:** 10.3390/polym14204278

**Published:** 2022-10-12

**Authors:** Felipe Robles-González, Teresa Rodríguez-Hernández, Antonio S. Ledezma-Pérez, Ramón Díaz de León, Marco A. De Jesús-Téllez, Héctor Ricardo López-González

**Affiliations:** Centro de Investigación en Química Aplicada, Blvd. Enrique Reyna Hermosillo 140, Saltillo 25294, Coahuila, Mexico

**Keywords:** ROP, biodegradable polyesters, TBD, organic catalyst

## Abstract

Three series of polyesters based on monomer combinations of *ε*-caprolactone (*ε*-CL), ethylene brassylate (EB), and l-Lactide (LLA) with the alkyl substituted lactone *ε*-decalactone (*ε*-DL) were synthesized at different molar ratios. Copolymers were obtained via ring opening polymerization (ROP) employing TBD (1,5,7-triazabicyclo-[4.4.0]-dec-5-ene), an organic catalyst which can be handled under normal conditions, avoiding the use of glove box equipment. The molar monomer composition of resulting copolymers differed from theoretical values due to lower *ε*-DL reactivity; their *M_n_* and *M_w_* values were up to 14 kDa and 22.8 kDa, respectively, and distributions were (*Ɖ*) ≤ 2.57. The thermal stability of these materials suffered due to variations in their *ε*-DL molar content. Thermal transitions such as melting (*T_m_*) and crystallization (*T_c_*) showed a decreasing tendency as *ε*-DL molar content increased, while glass transition (*T_g_*) exhibited minor changes. It is worth mentioning that changes in monomer composition in these polyesters have a strong impact on their thermal performance, as well as in their crystallization degree. Consequently, variations in their chemical structure may have an effect on hydrolyic degradation rates. It should be noted that, in future research, some of these copolymers will be exposed to hydrolytic degradation experiments, including characterizations of their mechanical properties, to determine their adequacy in potential use in the development of soft medical devices.

## 1. Introduction

One of the main research activities in health sciences is the development of biodegradable and biocompatible materials for medical applications. At present, surgical stents and other prosthetics are made of stainless steel, silver and platinum [1]. The use of metals in the construction of implants provides durability and mechanical properties. However, these medical devices suffer from limited control of their degradation rates, resulting in undesirable modifications, compromising functionality and causing adverse systemic reactions in the host [2]. To tackle the limitations of metals in the medical field, the introduction of polymers has brought solutions in terms of biocompatibility and biodegradability issues. Among such materials, aliphatic polyesters have been in the spotlight in recent years [3,4,5].

Aliphatic polyesters are characterized by their saturated linear structure and the presence of ester groups in their backbone. They are an interesting alternative in biomedical applications due to their renewable nature, physicochemical, thermal and morphological properties, ease of synthesis, processability and customizable degradation rates [6]. Current medical applications of polyesters include, but are not limited, tissue scaffolding, drug delivery systems and bone regeneration. Poly(glycolic acid) (PGA), poly(l-lactide) PLLA and poly(*ε*-caprolactone) (PCL) are examples of commonly synthesized aliphatic polyesters with adequate chemical, thermal and mechanical properties for the manufacturing of medical devices [5,7]. In tissue engineering, PLLA and PCL are frequently applied in bone, nerve, vascular and skin implants [8], while polyesters with high biodegradability, such as PGA and poly(*ε*-decalactone) (PDL), have been employed as encapsulations for proteins and antibiotics in drug delivery systems [9].

In ester copolymerization, materials with tailored features can be obtained by combining monomers with specific properties. For instance, inclusion of branched lactones comonomers as *ε*-decalactone into PLLA which possesses features as brittleness and low thermal stability, result in copolymers with improved mechanical properties, and higher degradation temperatures in comparison with PLLA homopolymer. These properties enhancements are mainly supported in the morphological changes induced by *n*-butyl side groups presented in PDL segments [10]. Regarding copolymers based on l-lactide/*ε*-caprolactone and ethylene brassylate/*δ*-valerolactone, variations in molar ratios of monomer were studied in order to establish a correlation between chemical composition, thermal properties and morphology. Copolymers based on l-lactide/*ε*-caprolactone exhibit enhanced thermal stability using PCL at 50–60 %mol, but their amorphous arrangement disrupts crystal formation [11]. Jin et al. [12] established a *T_m_* eutectic point in ethylene brassylate/*δ*-valerolactone random copolymers, wherein formulation with δ-valerolactone at 80 %mol induced co-crystallization and isodimorphism. Another example of biodegradable polyesters (*M_w_* up to 285 kDa) includes the use of biobased monomers, such as ethylene brassylate-*b*-*δ*-hexalactone, wherein a branched comonomer favors an amorphous arrangement, which has an impact on the mechanical properties, giving rise to possible applications in cell culture scaffolds [13]. On the other hand, triblock copolymers consisting of ethylene oxide-*b*-*ε*-caprolactone-*b*-γ-butyrolactone and *ε*-caprolactone-*b*-ethylene adipate-*b*-*ε*-caprolactone have also been reported; these synthesized copolyesters with *M_n_* up to 12.3 and 23.2 kDa, respectively, offer drug delivery system capabilities with specific drug-release profiles [14,15].

There are two main synthesis routes to obtain polyesters: polycondensation (step-growth polymerization) and ring-opening polymerization (ROP). In the first one, the polymerization reaction involves the esterification of diacids and diols, linking both types of molecules through the formation of ester groups. Although this type of polymerization can be performed under moderate conditions, and the required precursors are widely available, one of its most notorious disadvantages is the generation of byproducts, which promote reversible polymer–monomer reactions if they are not eliminated from the reaction media, leading to polymers with low molecular weights and wide dispersity [16]. Taking into consideration the limitations of polycondensation, ROP emerges as an alternative methodology to synthesize aliphatic polyesters which is based in the opening of cyclic monomers (lactones) by usage of catalysts, initiators or specific reaction conditions to obtain linear macromolecules. In contrast to step-growth polymerization, byproducts are not generated through ROP, allowing the synthesis of polymers with high molecular weights and narrow dispersity [17]. For instance, successful ROP of *ε*-caprolactone, *ε*-decalactone and pentadecalactone employing a zinc complex with phenoxy-imine ligand as a catalyst has been reported, achieving molecular weights at 21–73 kDa and dispersities of 1.8–2.3 [18]. In addition, some researchers have reported the implementation of stannous octoate and bismuth salts as catalyst in the synthesis of PLLA, PDL and l-lactide/*ε*-decalactone copolymers with block and random architectures via ROP methodologies, in which molar masses reached 58.7 kDa for PLLA, 86.6 kDa for PDL and 54.1 kDa for l-lactide/*ε*-decalactone copolymers with dispersity values of 1.2–2.2, although reaction times were higher than 48 h and temperatures were up to 130 °C [10,19]. 

It is worth mentioning that ROP via organometallic-catalysis represents a feasible technique for aliphatic polyester synthesis of, e.g., PCL and PLA with high values of conversions and molar mass, as well as narrow dispersion [20]; however, some of the major drawbacks are the purity conditions for handling the organometallic inititator and monomers, and the absence of oxygen and environmental humidity in the reaction media to ensure high reaction performance [10,21]. Furthermore, ineffective and expensive purification procedures of the synthesized polymers are required for the removal of metal complex residues coming from catalysts like stannous octoate (Sn(Oct)_2_), bismuth salts and neodymium isopropoxide. For instance, FDA regulations state that the presence of Sn(Oct)_2_ cannot exceeded 20 ppm, and so usage of this kind of reagent may be result hazardous in medical, pharmaceutical and food packaging applications [22]. Other drawbacks to consider are depolymerization and oxidation reactions, which increase the toxicity, lower the biocompatibility, and influence the chemical, thermal and mechanical properties [23]. To overcome the consequences of organometallic initiators, polyester synthesis via metal-free ROP is a green and low-cost alternative, featuring milder reaction conditions and undemanding purification techniques of raw materials and products. In this regard, 1,5,7-triazabicyclo[4.4.0]dec-5-ene (TBD) and 1,8-diaza[5.4.0]bicycloundec-7-ene (DBU) have emerged as organo-catalytic options providing versatility in terms of the monomer/catalyst and initiator/catalyst ratios, control over molecular weight and dispersity, ease of storage, handling without requirements of inert atmosphere conditions and consistent biocompatibility due to their easy removal [24]. Therefore, TBD and DBU catalysts are interesting candidates for the synthesis of PLA, PCL, PDL and PEB; previous works have employed these reaction systems for the synthesis of these polyesters, reaching molecular weight values from 2.4 to 85 kDa and dispersities of 1.05–1.90 in periods shorter than 24 h [10,13,25].

In this work, series of polyesters based on combinations of L-lactide/*ε*-decalactone, ethylene brassylate/*ε*-decalactone and *ε*-caprolactone/*ε*-decalactone at different molar ratios were achieved through the ROP methodology using TBD as a catalyst. It is worth mentioning that there are no previous works reporting the systematic study of the chemical, thermal and morphological properties of this type of copolymer and the influence of *ε*-decalactone on such properties. Therefore, these aliphatic polyesters may exhibit biodegradable features through variations in their chemical structures, possibly resulting in an interesting alternative for the development of soft medical devices.

## 2. Materials and Methods

### 2.1. Reagents

The monomers *ε*-caprolactone (*ε*-CL), *ε*-decalactone (*ε*-DL) and ethylene brassylate (EB) (Sigma-Aldrich) were purified via a distillation system which included Na^0^ as a drier reagent, a vacuum pressure at 15 mm Hg and a temperature of 160 °C to achieve complete removal of humidity and oxygen. L-Lactide (L-LA) (Sigma-Aldrich, St. Louis, MO, USA), was purified through a crystallization process into ethyl acetate (J. T. Baker, Radnor, PA, USA) solution (33.33% weight). Other reagents such as TBD (TCI chemicals, Portland, OR, USA), benzyl alcohol and trioxane (Sigma-Aldrich, St. Louis, MO, USA) were used as received. Toluene and methanol were provided by J. T. Baker. Toluene was washed with H_2_SO_4_, dried in CaCl_2_, stirred under reflux conditions with LiAlH_4_ and distilled using a sodium/benzophenone complex, while methanol was used as received.

### 2.2. Characterization and Equipment

The chemical composition was determined by proton nuclear magnetic resonance (^1^H NMR) at room temperature using a Bruker (Billerica, MA, USA) Advance III 400 MHz spectrometer and deuterated chloroform (CDCl_3_) as a solvent. Additionally, characteristic functional groups in copolymers were confirmed through Fourier transform infrared spectroscopy (FTIR)–attenuated total reflectance (ATR) mode, recorded from 4000 to 400 cm^−1^ in a FTIR spectrometer model Nicolet iS5 from Thermo-Scientific, (Madison, WI, USA). Gel permeation chromatography (GPC) was used in the determination of molar mass (*M_n_* and *M_w_*) and distribution (*Ɖ*) in copolymers employing an Agilent ( Santa Clara, CA, USA) series 1100 calibrated with polystyrene standards, CHCl_3_ (HPLC grade) as eluent, a refraction index detector, a set of three columns PL-gel 5 µm, one column 10^6^ Å and two column Mixed-C. Thermal stability was recorded in a thermogravimetric analyzer Q500 model from TA Instruments (New Castle, DE, USA); the heating rate was 10 °C min^−1^ from 30 to 600 °C under N_2_ atmosphere. Thermal transitions (*T_g_*, *T_c_* and *T_m_*) were obtained with a differential scanning calorimeter (DSC–2500 from TA Instruments) at heating and cooling rates of 10 °C min^−1^ from −70 to 100 °C under a N_2_ atmosphere. X-ray diffraction (XRD) provides information about the crystalline arrangements in copolymers; XRD analyses were performed using a Bruker D8 Advance ECO. The radiation frequency selected was the k_α1_ line from Cu (1.5406 Å). A power supply of 40 kV and 25 mA and an angle from 3 to 110° with an increment rate of 0.02° at 0.5 s were used.

### 2.3. Polyester Copolymers Synthesis

**Scheme 1:**
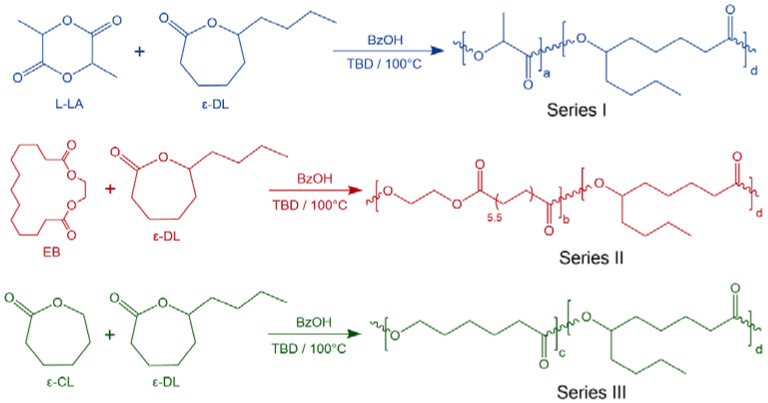
Synthesis of Series I–III via organocatalytic ROP.

Polyesters were synthesized via ROP using cyclic esters monomers with alkyl side groups (*ε*-DL) and non-substituted (*ε*-CL, EB, L-LA). In Series I, the combination of monomers was L-LA and *ε*-DL. Meanwhile, Series II was done by EB and *ε*-DL and Series III by *ε*-CL and *ε*-DL (see Scheme 1). It should be noted that the chemical arrangement in these copolymers was block architecture, taking into consideration the sequential addition of monomers. In more detail, the first step corresponded to the addition of *ε*-DL. The non-substituted monomer was added afterwards, following the periods of time mentioned in the Supplementary Information section (Tables S1–S3). Polymerization reactions were done in a 50 mL Schlenk flask, wherein *ε*-DL, catalyst (TBD), NMR reference (trioxane) and solvent (toluene) were added at the corresponding molar ratios (Tables S1–S3). Each reaction mixture was degassed through three vacuum/N_2_ cycles to eliminate oxygen and humidity traces. Thereafter, the reaction system was exposed to heating (100 °C) and stirring (400 rpm) and the initiator (benzyl alcohol, BzOH) was added dropwise. It is worth mentioning that the second monomer (L-LA, EB, *ε*-CL) was added, taking into consideration the *ε*-DL percentage loaded in each formulation. The established time for all reactions was fixed at 24 h. Finally, the purification process consisted of the evaporation of toluene, washing the product twice in cold methanol (~0 °C) to eliminate raw materials, filtration and drying in a vacuum oven at 40 °C in a period of 24 h. The reaction conditions implemented in this work come from previous studies, although parameters such as the catalyst, reaction time, and molar ratios were modified [26].

## 3. Results

### 3.1. Polyester Copolymers Synthesis 

**Figure 1:**
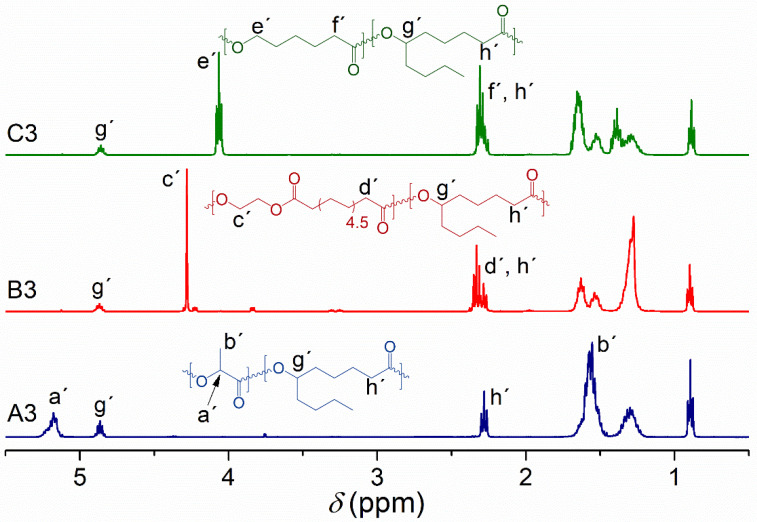
^1^H NMR spectra of representative polyesters A3, B3 and C3.

Monomer conversion and the chemical structure in the copolymer series were studied by ^1^H NMR in order to corroborate the expected signals and chemical shifts (*δ*), as well as the percentages of monomers in the synthesized copolymers. The success of the ROP reactions catalyzed by TBD was monitored by changes in *δ* of representative protons in polymers and monomers, specifically, proton signals in methylene and methine groups, located in the α position of O-C=O (carboxylic groups). Moreover, calculated conversions for the homopolymerization of the four studied monomers were >90% after 24 h, which indicated the high catalytic efficiency of TBD in these polymerizations due to its ability to act as a H-bond donor and acceptor, granted by the secondary and tertiary nitrogen atoms in its structure, thereby favoring the ring-opening mechanism of cyclic esters [27]. Such conversions in the given duration represent an improvement over the reaction time established by Ramos-Durán et al., who employed a neodymium isopropoxide-catalyzed system for the polymerization of *ε*-CL and *ε*-DL, among other lactones [28]. Figure 1 shows ^1^H NMR spectra of copolymers A-3, B-3 and C3. Likewise, the ^1^H NMR spectra of monomers, as well as those of the rest of the copolymers in Series I–III, are displayed in the Supplementary Materials (Figures S1–S4). In the Series I spectra, the target PLA proton CH *α* O-CO was fixed at 5.18 ppm. Regarding PDL segments in the copolymers of series I–III, the characteristic proton in the methine group was located at 4.86 ppm, resulting in *δ* matching the expected values for the proton CH *α* O-CO in both the PLA and PDL segments. It is worth to mention that ROP in L-LA with TBD as a catalyst and the applied reaction conditions favor the obtention of atactic PLA, which could develop an amorphous arrangement (see Section 3.2). Additionally, the ^1^H NMR spectra in Series I (Figure S2) reveal multiple signals from 5.27 to 5.14 ppm, suggesting the presence of several stereoregular arrangements. These studies were mentioned by Moins et al., who achieved the synthesis of isotactic PLA via ROP using a TBD catalyst under cryogenic conditions (−72 °C), while non-stereoregular PLA was obtained at 23 °C [29]. 

**Figure 2:**
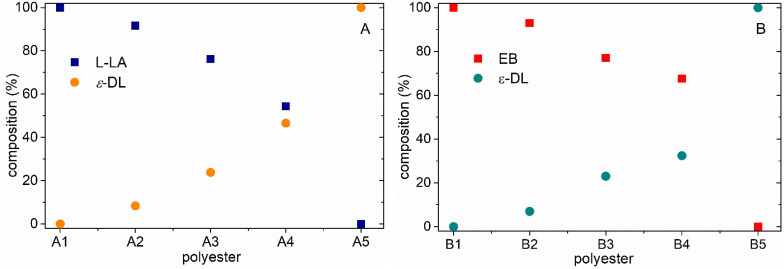

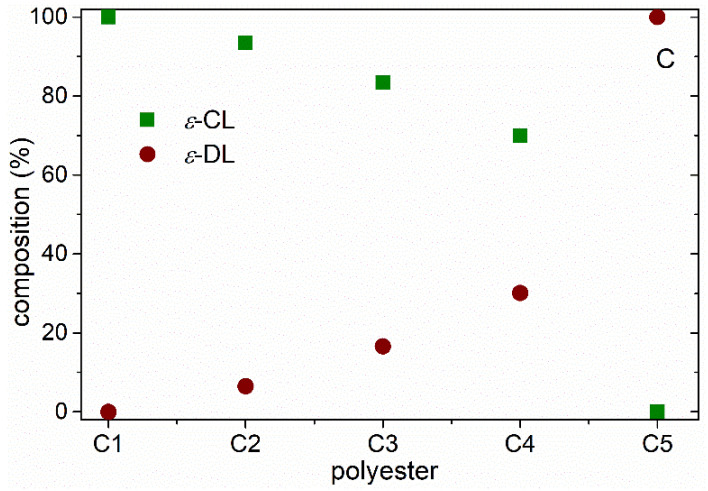
Percentage composition of comonomers determined through ^1^H NMR in Series I (**A**), Series II (**B**) and Series III (**C**).

CH_2_ *α* O-CO of PEB segments in Series II and PCL segments in Series III showed the expected *δ* of 4.28 ppm and 4.06 ppm, respectively, for these polyesters. A composition analysis was performed, taking as reference the integration values of the signals previously mentioned. The calculation methodology employed was previously described by Ramos-Durán and co-workers [28]. The results are displayed in Figure 2 and Table 1, Table 2 and Table 3. Notably, the molar mass percentage of *ε*-DL was less than the stoichiometric reference; this was attributed to the minor reactivity ratio in comparison to the non-substituted lactones (*ε*-CL, L-LA and EB) [30].

**Figure 3:**
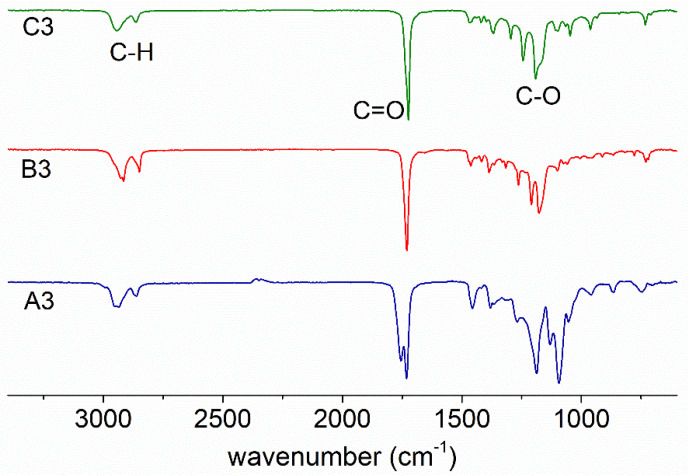
Absorption bands of characteristic functional groups through FTIR in A3, B3 and C3.

The spectra of polyesters A3, B3 and C3 are shown in Figure 3. A complete FTIR characterization of Series I–III is presented in the Supplementary Materials (Figures S5–S7). The characteristic stretching absorption bands for C-H were observed at ~2940 cm^−1^ (CH_3_), ~2860 cm^−1^ (CH_2_), while the carbonyl group was located at ~1730 cm^−1^ (C=O) and C-O absorption bands exhibited a wide range of wave number, i.e., from 1244 to 1093 cm^−1^. We draw your attention to the A3 spectrum, in which two absorption bands were observed in the C=O region. Firstly, the signal which corresponded to C=O in PLA was fixed at 1750 cm^−1^, while the C=O groups related with PDL segments were observed at 1730 cm^−1^. The overlap in the absorption bands was related to differences in the chemical environment of PLA and PDL, wherein carbonyl groups in PLA underwent a bathochromic effect due to vicinal interactions between polar groups (O-C=O), in contrast with PDL, in which there were longer distances between these groups [31].

**Figure 4:**
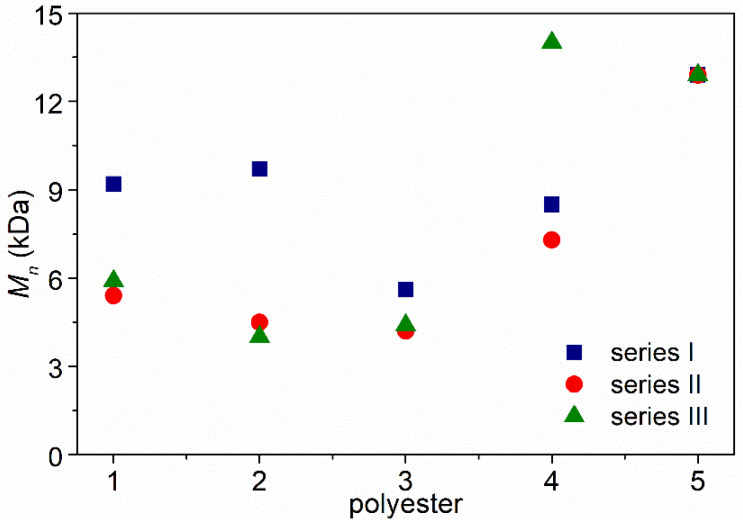
Average number molar mass (*M_n_*) in Series I–III.

**Figure 5:**
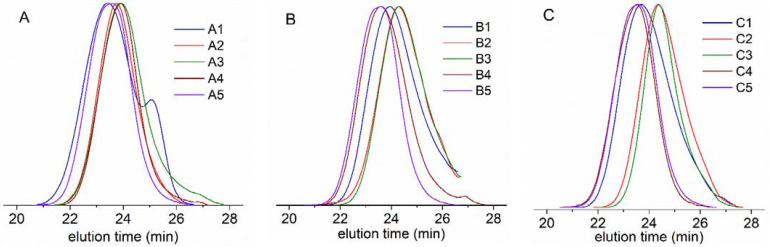
GPC analysis of polyesters of Series I (**A**), Series II (**B**) and Series III (**C**).

Molar mass analysis and the distributions of the obtained polyesters are shown in Table 1, Table 2 and Table 3. In Series I–III, the theoretical average molecular weight (*M_n_*) was calculated, ranging from 28.5 to 42.5 kDa (Tables S1–S3), while the experimental values of *M_n_* and *Ɖ* recorded by GPC gave *M_n_* ranging 4.0 to 14.0 kDa, as shown in Figure 4, denoting that the target molar masses were not achieved through the performed ROP organocatalytic reactions. Taking into consideration the temperature (100 °C) and time established for all reactions (24 h), the low molecular weights were attributed to secondary transesterification reactions which took place during the polymerization of monomers that showed higher reactivity than *ε*-DL, especially L-LA and *ε*-CL, leading to the generation of polymeric chains with reduced lengths and high dispersities [32]. Therefore, in Series I and III, a narrowing tendency in the dispersities was observed as the *ε*-DL content increased. It should be mentioned that these results are approximative, given that the measurements were carried out using polystyrene standard references, which possess a different chemical nature than polyester-based systems. Likewise, chromatograms obtained from GPC allowed us to observe that the molar mass distributions had acceptable monomodal performance (*Ɖ* ≥ 1.63) in all ROP reactions (Figure 5) [33]. In contrast, PLA homopolymer (A-1) showed the formation of an oligomer, observed as a bimodal distribution. This was attributed to the reaction conditions, wherein loss of control in the propagation step was induced, or by the presence of intermolecular transesterification reactions [34] due to long reaction periods (24 h). The sequential addition of the second monomer (L-LA, EB, *ε*-CL) at specific times, as shown in Tables S1–S3, led us to expect a block arrangement in the copolymers. Through the analysis of the resulting chromatograms, monomodal curves suggested that this sequential addition did not result in the formation of oligomers or low molar mass species. 

### 3.2. Thermal and Morphological Analysis

Thermal stability in Series I–III was measured to determine the degradation profiles (Supplementary Materials, Figures S8–S10) of these compounds. Data are reported in Table 1, Table 2 and Table 3, taking a 5% weight loss as a reference. The copolymers in Series I exhibited the lowest stability, in a range from 203 to 237 °C, in comparison to Series II and III (294–324 °C). It should be mentioned that Series I contained PLA blocks, wherein carboxylic groups are closer to each other in comparison to PEB, PCL and PDL blocks. As such, higher quantity of O=C-O groups favored chains scission and the formation of CO_2_ as a byproduct at lower temperatures [10]. Likewise, thermal stability is important for setting heating–cooling temperatures in DSC analyses, making it possible to perform tests without compromising the chemical structure of the polyesters.

**Figure 6:**
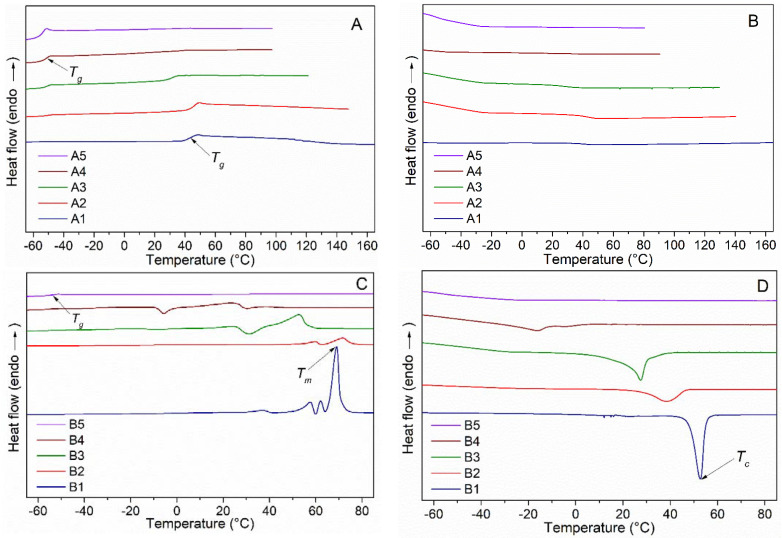

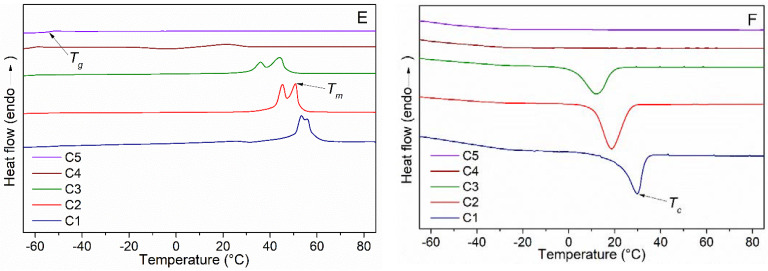
Thermograms of Series I–III. (**A**) heating-Series I; (**B**) cooling-Series I; (**C**) heating-Series II; (**D**) cooling-Series II; (**E**) heating-Series III; (**F**) cooling-Series III.

Regarding DSC, analyses were recorded from −70 to 170 °C in Series I and −70 to 100 °C in Series II and III. Some differences in thermal transitions values were observed during the heating and cooling processes, as shown in Figure 6 and Table 1, Table 2 and Table 3. For instance, A1-A5 only exhibited second order transitions (*T_g_*), possibly due to the amorphous arrangement in the PLA and PDL blocks (Figure 6A,B). It should be noted that the polyesters in Series I were synthesized at 100 °C, which would have a detrimental effect on the crystallinity of PLA. This fact is supported by the reports mentioned in Section 3.1, where, for example, the PLA obtained using TBD as catalyst at a temperature of 23 °C developed an atactic arrangement [29]. In addition, some works related to block copolymers based on L-LA and *ε*-DL observed a similar thermal performance, as well as loss of crystalline order caused by microphase separation and perturbation of chain interactions due to side butyl groups in PDL segments [21,30].

In Series II, B1 exhibited endotherms at 57, 62 and 68.8 °C (Figure 6C) that may be related to semicrystalline arrangements. Additionally, the multiplicity in transitions could be associated with differences in the lamellar thickness [35]. This thermal performance could be supported in the structural features of EB, taking in consideration its 11 methylene units between carboxylic groups, and so, the macromolecules can acquire several arrangements that are function of carboxylic interaction and stability in the backbone conformation Likewise, B2–B4 exhibited endotherms in a wide temperature range, while B3 and B4 developed melt recrystallization events, in which the presence of a branched comonomer (*ε*-DL) had an impact on the crystallization decrease due to decoupled intermolecular interactions. B5 (PDL homopolymer) only displayed *T_g_* (−55.1 °C), which is associated with amorphous domains.

In series III, C1–C3 exhibited endothermic transitions composed of two broad peaks that may have been related to different crystalline arrangements (Figure 6E). These transitions could have a correlation with ordered lamellar domains in the PCL blocks (higher temperature peak) in coexistence with backbone segments that suffer from reduced crystallinity due to their reduced interactions and chain folding [36]. It is worth mentioning that the percentage of *ε*-DL ≥ 30% mol was sufficient to achieve a transition from semi-crystalline to amorphous arrangement, as shown in C4. These morphological changes are of interest for biodegradable applications, where control over degradation rates is required.

**Figure 7:**
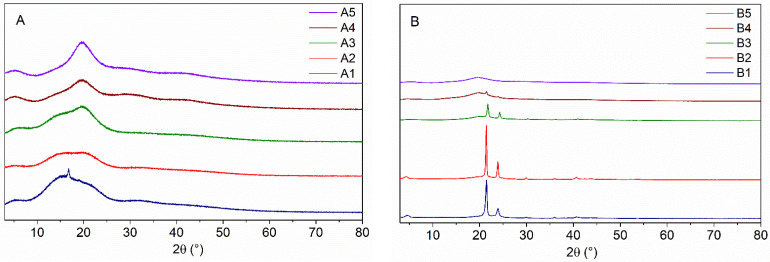

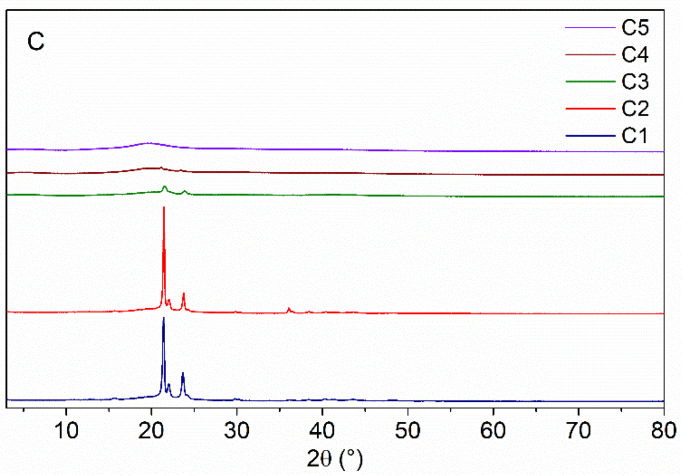
Diffractograms in Series I (**A**), Series II (**B**) and Series III (**C**).

Regarding the morphological features in Series I–III, XRD patterns were collected to elucidate the structural organization. For instance, diffractograms in Series I exhibited broad undefined peaks in region 2θ = 10–30°, characteristic of non-ordered domains. This amorphous arrangement was predicted in formulations based on L-LA:*ε*-DL (Figure 7A). In the case of Series II and III, polyesters with low percentages of *ε*-DL developed a semicrystalline arrangement with similar diffractograms and 2θ values (Figure 7A,B). Herein, they exhibited a main sharp peak at 2θ = 21.45°, corresponding to reflection (110), and a shorter signal at 2θ = 23.8°, corresponding to reflection (200) [37,38]. Additionally, an increase of *ε*-DL in formulations led to an intensity reduction in the diffraction peaks, denoting a loss of crystalline domains in the polyesters.

**Table 1:** Comonomer molar percentage, *M_n_*, *M_w_*, *Ɖ*, thermal properties and *X_c_* in Series I.

Series I	L-LA (% mol)	*ε*-DL (% mol)	*M_n_* (kDa)	*M_w_* (kDa)	*Ɖ*	*T_m_* (°C)	*T_c_* (°C)	*T_g_* (°C)	*TGA* (5%w)	^c^ *X_c_*
A-1	100	0	9.2	20.8	2.26	-	-	43.1	203	-
A-2	91.6	8.4	9.7	15.9	1.64	-	-	^a^ −51.5, ^b^ 45	247	-
A-3	76.2	23.8	5.6	13.6	2.42	-	-	−51.7, 30	217	-
A-4	54.4	46.6	8.5	14.8	1.74	-	-	−53.2	237	-
A-5	0	100	12.9	21.7	1.68	-	-	−55.1	316	-

^a^*T_g_* in PDL; ^b^ *T_g_* in PLA; ^c^ *X_c_* obtained via integration peaks from XRD diffractograms (Figure 7).

**Table 2:** Comonomer molar percentage, *M_n_*, *M_w_*, *Ɖ*, thermal properties and *X_c_* in Series II.

Series II	EB (% mol)	*ε*-DL (% mol)	*M_n_* (kDa)	*M_w_* (kDa)	*Ɖ*	*T_m_* (°C)	*T_c_* (°C)	*T_g_* (°C)	*TGA* (5%w)	*X_c_*
B-1	100	0	5.4	12.6	2.34	68.8	52.7	-	294	1.0
B-2	93.0	7.0	4.5	9.9	2.2	71.8	38.5	-	321	0.90
B-3	77.0	23.0	4.2	8.9	2.12	52.6	27.5	−56.8	316	0.45
B-4	67.6	32.4	7.3	17.8	2.44	23.5	−16.1	−56.3	318	-
B-5	0	100	12.9	21.7	1.68	-	-	−55.1	3.16	-

**Table 3:** Comonomer molar percentage, *M_n_*, *M_w_*, *Ɖ*, thermal properties and *X_c_* in Series III.

Series III	*ε*-CL (% mol)	*ε*-DL (% mol)	*M_n_* (kDa)	*M_w_* (kDa)	*Ɖ*	*T_m_* (°C)	*T_c_* (°C)	*T_g_* (°C)	*TGA* (5%w)	*X_c_*
C-1	100	0	5.9	15.2	2.57	55.8	29.8	-	324	1.0
C-2	93.5	6.5	4.0	8.2	2.04	50.8	18.6	-	317	1.01
C-3	83.4	16.6	4.4	7.8	1.78	44.1	12.1	−61.7	317	0.25
C-4	69.9	30.1	14	22.8	1.63	21.5	-	−62.1	319	-
C-5	0	100	12.9	21.7	1.68	-	-	−55.1	316	-

## 4. Conclusions

In this work, the synthesis of three series of polyesters using a combination of an alkyl substituted monomer (*ε*-DL) with other non-susbstituted monomers (L-LA, EB, *ε*-CL) was achieved. The architecture of the copolymers was induced block-type, taking into consideration the sequential addition of monomers. TBD, an organic catalyst, was successfully implemented in ROP reactions without usage of anhydrous handling conditions. Therefore, this methodology may be implemented with other monomers, avoiding the requirement of glove box equipment.

In these copolymers, a reduction of crystallinity was observed as result of an increase in the molar content of *ε*-DL. In particular, at molar concentrations ≥30%, the remains of crystalline domains were embedded into the amorphous domains. The ROP of L-LA under the established reaction conditions resulted in an amorphous polyester, despite the usage of a reagent with specific stereoregularity.

Generally speaking, the crystallization features of these copolymers can be modified with branched comonomer inclusion in the backbone of the polyesters. Therefore, it is suggested that the degradation rates of this class of materials can be modified by changes in the chemical arrangement of their backbone. In addition, some of the studied polyesters are currently being analyzed under hydrolytic degradation conditions, and encouraging results are being obtained. Likewise, our copolymers are of potential value for the development of soft medical devices with biodegradability and biocompatibility features, as their synthesis does not involve the use of organometallic reagents.

## Data Availability

Not applicable.

## Supplementary Materials

The following supporting information can be downloaded at: https://www.mdpi.com/article/10.3390/polym14204278/s1, Table S1. Nomenclature and molar ratios in synthesis of Series I; Table S2. Nomenclature and molar ratios in synthesis of Series II; Table S3. Nomenclature and molar ratios in synthesis of Series III; Figure S1. ^1^H NMR spectra of monomers; Figure S2. ^1^H NMR spectra in polyesters of Series I; Figure S3. ^1^H NMR spectra in polyesters of Series II; Figure S4. ^1^H NMR spectra in polyesters of Series III; Figure S5. FTIR-ATR spectra in polyesters of Series I; Figure S6. FTIR-ATR spectra in polyesters of Series II; Figure S7. FTIR-ATR spectra in polyesters of Series III; Figure S8. TGA thermograms in polyesters of Series I; Figure S9. TGA thermograms in polyesters of Series II; Figure S10. TGA thermograms in polyesters of Series III.
