# Supplementary material for: Development of Biodegradable Polyesters: Study of Variations in Their Morphological and Thermal Properties through Changes in Composition of Alkyl-Substituted (ε-DL) and Non-Substituted (ε-CL, EB, L-LA) Monomers
Source: Polymers (Basel). 2022 Oct 12;14(20):4278. doi: 10.3390/polym14204278 (PMC9612037; doi:10.3390/polym14204278)
Supplement: Supplementary file 1 [file polymers-14-04278-s001.zip › polymers-1946144-supplementary.pdf]

polymers

MDPI

Supporting information

Development of Biodegradable Polyesters: Study of Variations in Their Morphological and Thermal Properties through Changes in Composition of Alkyl-Substituted (ε-DL) and non-Substituted (ε-CL, EB, L-LA) Monomers

Felipe Robles-González, Teresa Rodríguez-Hernández, Antonio S. Ledezma-Pérez, Ramón Díaz de León, Marco A. De Jesús-Téllez * and Héctor Ricardo López-González *

Centro de Investigación en Química Aplicada, Blvd. Enrique Reyna Hermosillo140, 25294, Saltillo Coahuila, Mexico

* Correspondence: marco.tellez@ciqa.edu.mx (M.A.D.J.-T.); ricardo.lopez@ciqa.edu.mx (H.R.L.-G.)

Table S1. Nomenclature and molar ratios in synthesis of Series I.

Series I	L-LA (% mol)	ε-DL (% mol)	molar ratio $^1M_a : ^2M_d : ^3C : ^4I$	^5M_a (h)	$M_{n\ theo.}$ (kDa)
A-1	100	0	250 : 0 : 5 : 1	-	36.03
A-2	85	15	212 : 38 : 5 : 1	4	37.02
A-3	65	35	162 : 88 : 5 : 1	6	38.33
A-4	50	50	125 : 125 : 5 : 1	8	39.30
A-5	0	100	0 : 250 : 5 : 1	-	42.56

[1]M_a=L-LA; [2]M_d=ε-DL; [3]C=catalyst (TBD); [4]I=initiator (BzOH); [5]time for addition of M_a.

Table S2. Nomenclature and molar ratios in synthesis of Series II.

Series II	EB (% mol)	ε-DL (% mol)	molar ratio $^1M_b : ^2M_d : C : I$	^3M_b (h)	$M_{n\ theo.}$ (kDa)
B-1	100	0	125 : 0 : 5 : 1	-	33.80
B-2	85	15	106 : 38 : 5 : 1	4	35.13
B-3	65	35	81 : 88 : 5 : 1	6	36.88
B-4	50	50	63 : 125 : 5 : 1	8	38.31
B-5	0	100	0 : 250 : 5 : 1	-	42.56

[1]M_b=EB; [2]M_d=ε-DL; [3]time for addition of M_b.

Table S3. Nomenclature and molar ratios in synthesis of Series III.

Series III	ε-CL (% mol)	ε-DL (% mol)	molar ratio [1]M_c : [2]M_d : C : I	[3]M_c (h)	$M_{n\ theo}$ (kDa)
C-1	100	0	250 : 0 : 5 : 1	-	28.53
C-2	85	15	212 : 38 : 5 : 1	4	30.67
C-3	65	35	162 : 88 : 5 : 1	6	33.47
C-4	50	50	125 : 125 : 5 : 1	8	35.55
C-5	0	100	0 : 250 : 5 : 1	-	42.56

[1]*M_b=EB;* [2]*M_d=ε-DL;* [3]*time for addition of M_c.*

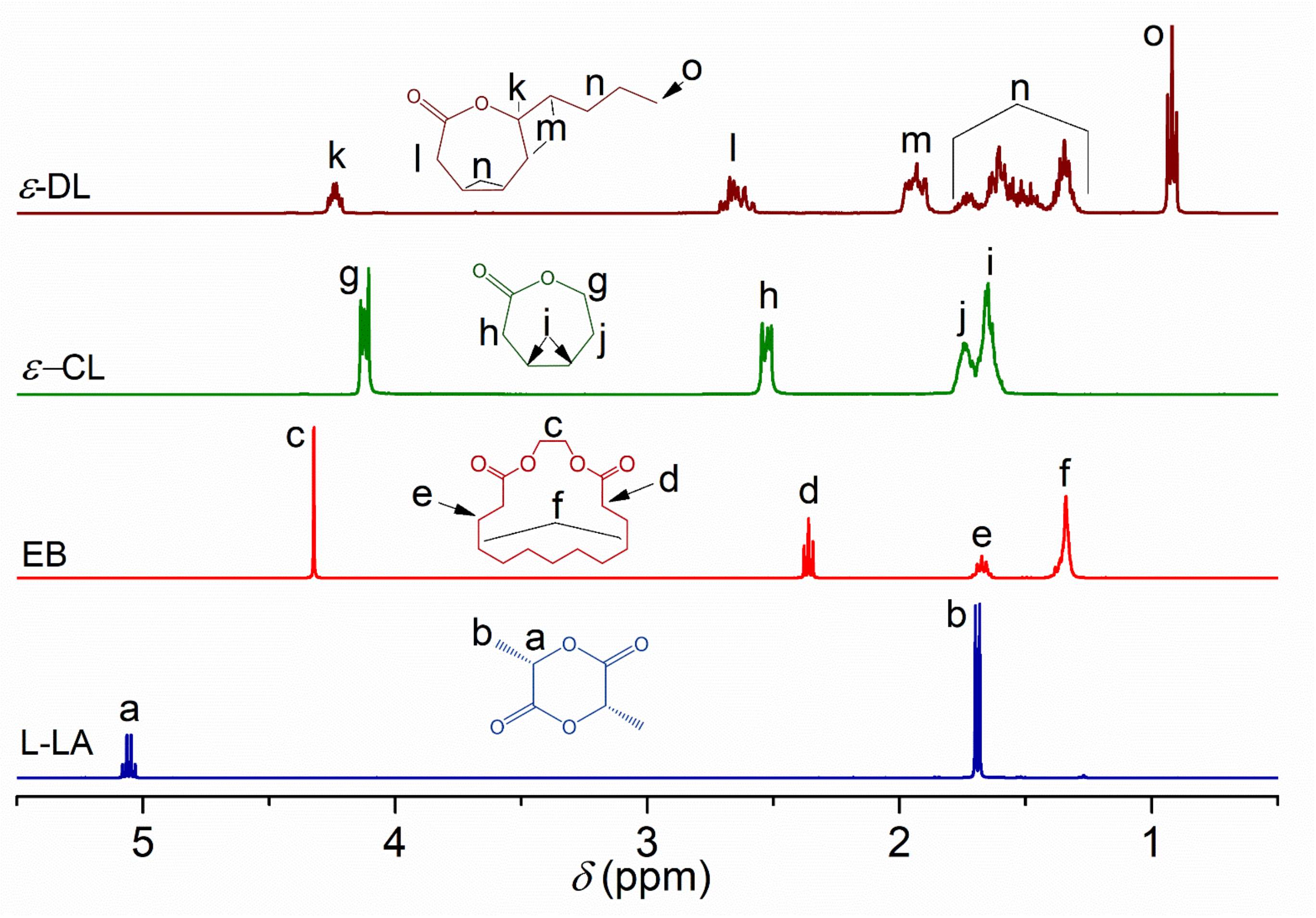

Figure S1. ^{1}H NMR spectra of monomers

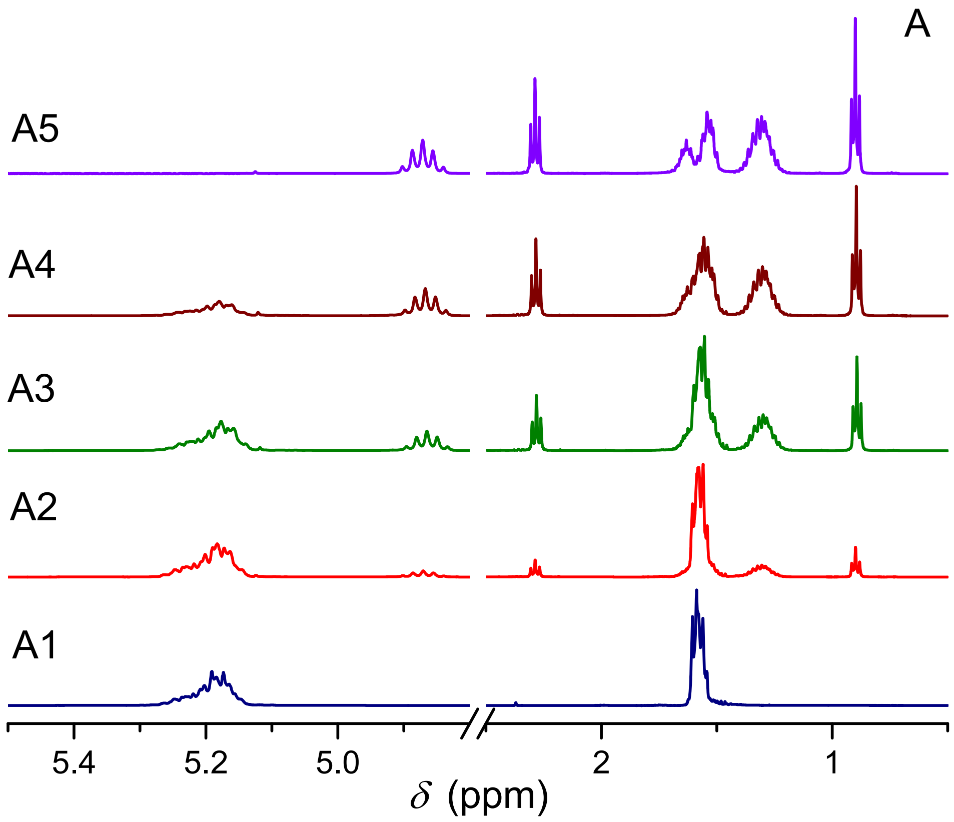

Figure S2. ¹H NMR spectra in polyesters of Serie I

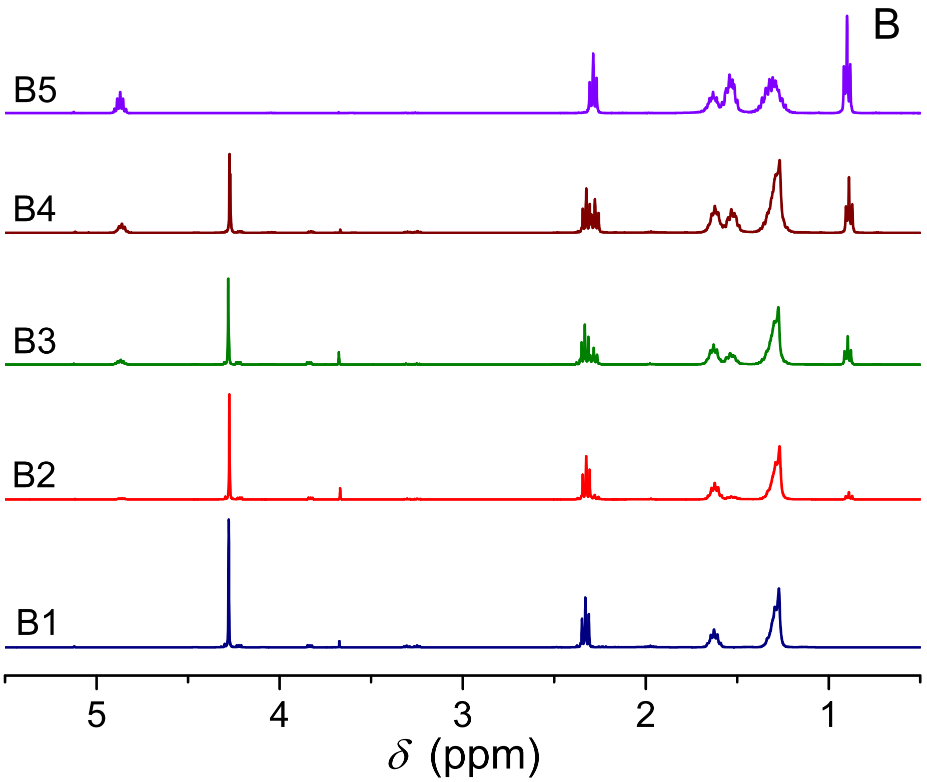

Figure S3. ¹H NMR spectra in polyesters of Serie II

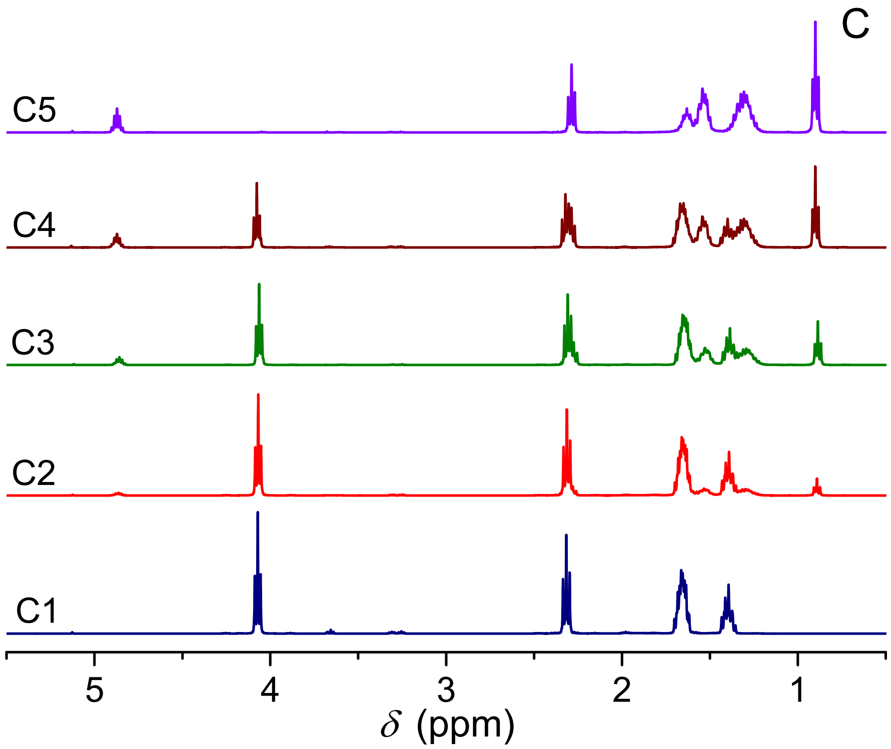

Figure S4. ¹H NMR spectra in polyesters of Serie III

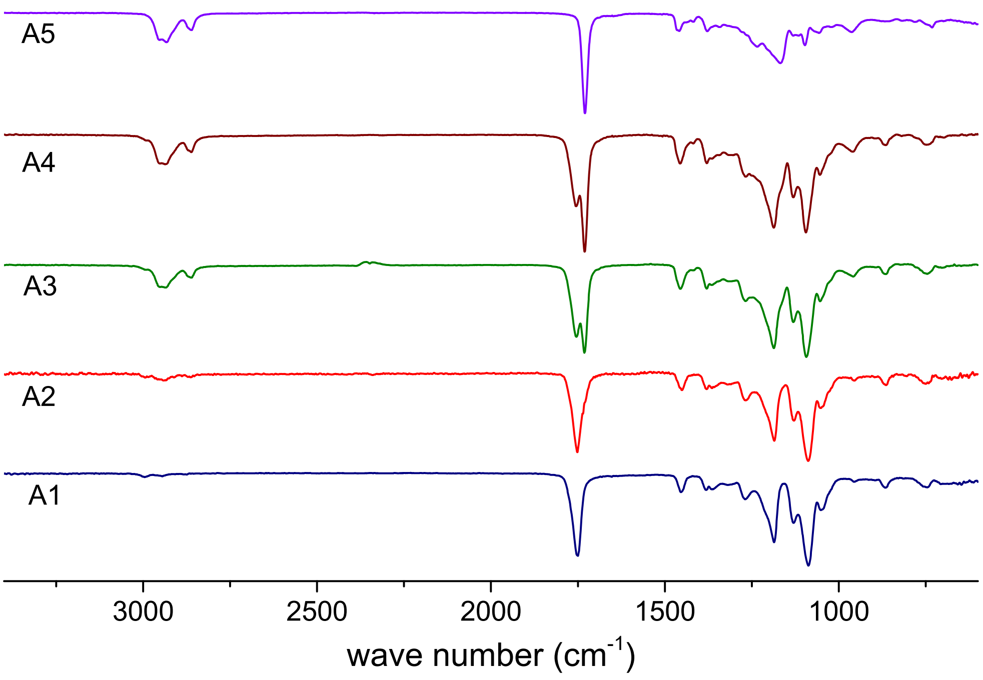

Figure S5. FTIR-ATR spectra in polyesters of Serie I

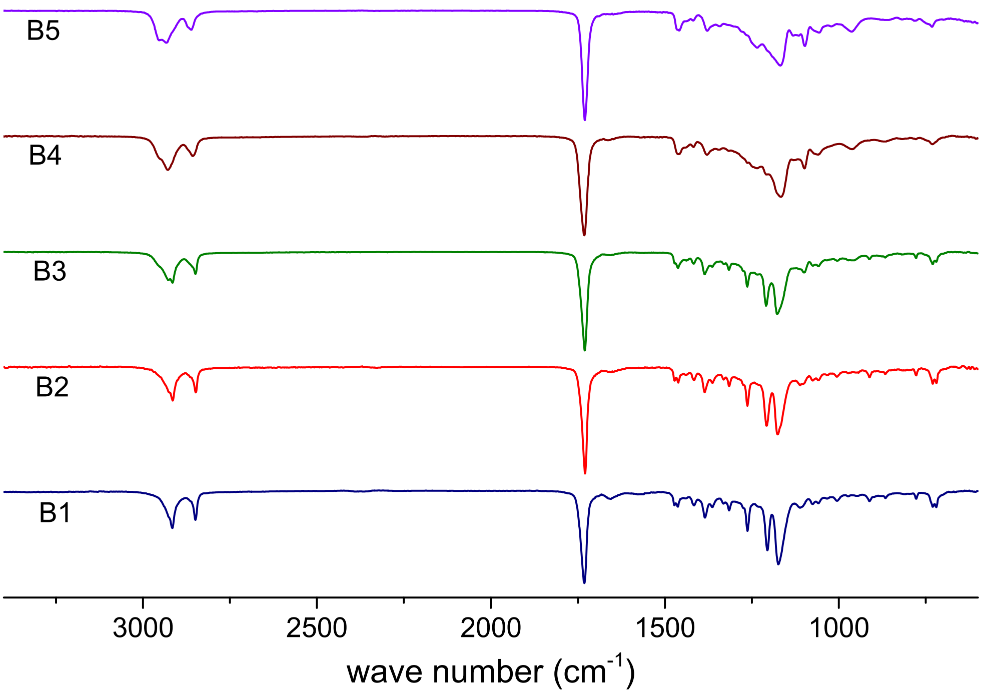

Figure S6. FTIR-ATR spectra in polyesters of Serie II

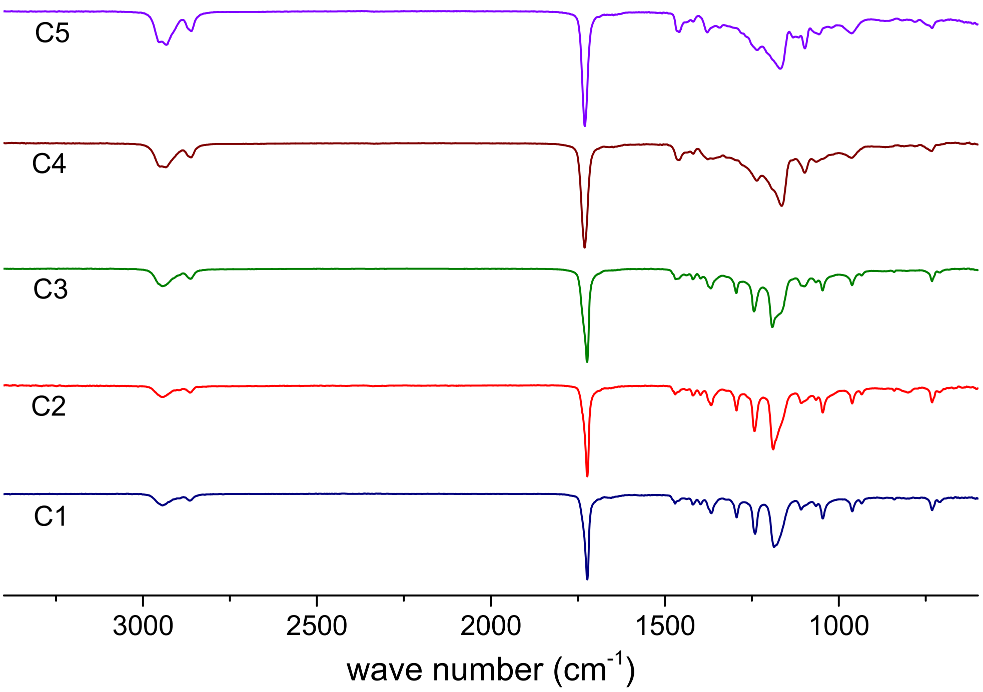

Figure S7. FTIR-ATR spectra in polyesters of Serie III

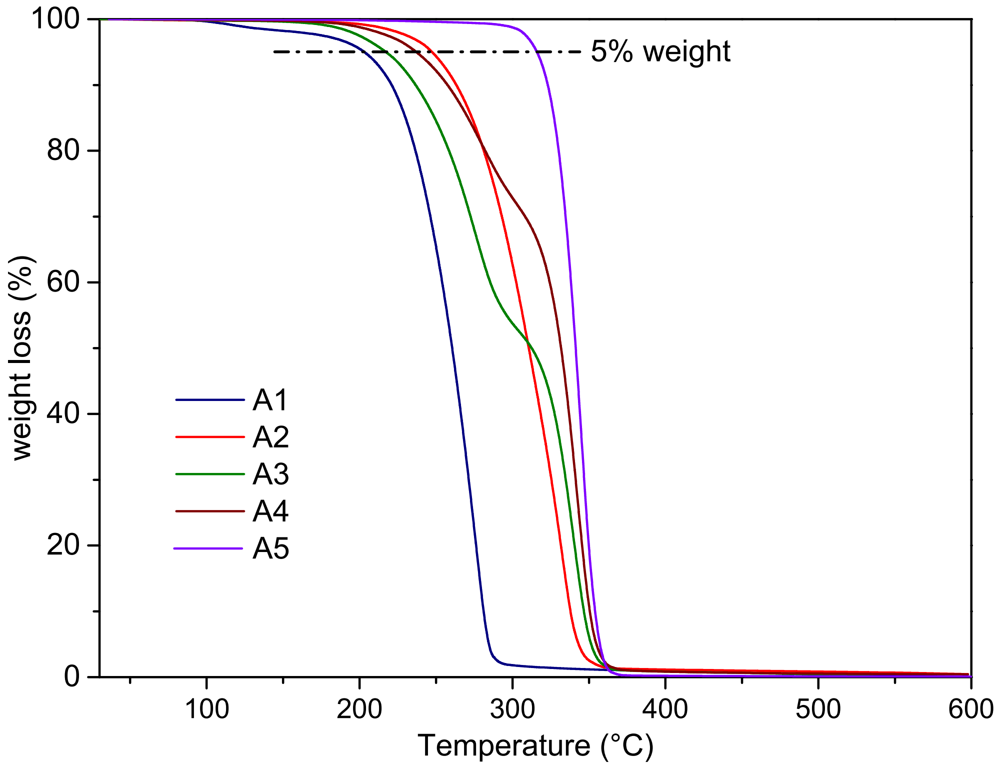

Figure S8. TGA thermograms in polyesters of Serie I

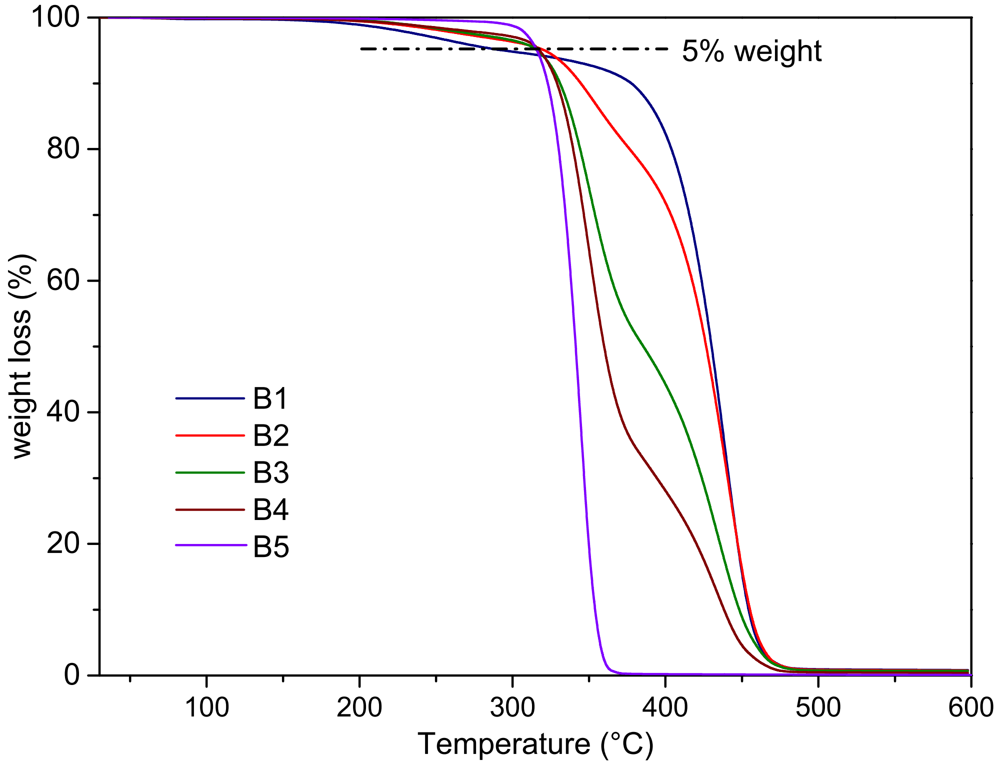

Figure S9. TGA thermograms in polyesters of Serie II

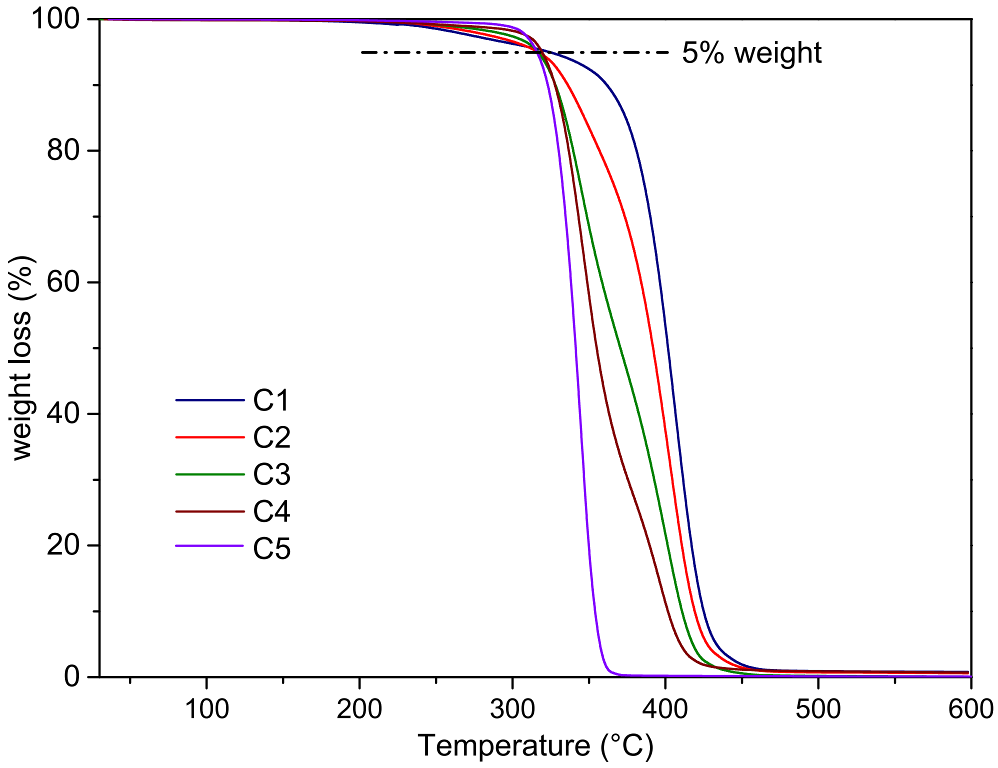

Figure S10. TGA thermograms in polyesters of Serie III
